# The changing epidemiology of VanB *Enterococcus faecium* in Poland

**DOI:** 10.1007/s10096-018-3209-7

**Published:** 2018-02-13

**Authors:** Ewa Sadowy, Iwona Gawryszewska, Alicja Kuch, Dorota Żabicka, Waleria Hryniewicz

**Affiliations:** 1 Department of Molecular Microbiology, National Medicines Institute, ul. Chełmska 30/34, 00-725 Warsaw, Poland; 2 Department of Epidemiology and Clinical Microbiology, National Medicines Institute, Chełmska 30/34, 00-725 Warsaw, Poland

**Keywords:** Epidemic lineage, Population shift, Transposon, Plasmid, Diversity

## Abstract

Increasing prevalence of VanB *Enterococcus faecium* in Polish hospitals reported to National Reference Centre for Susceptibility Testing (NRCST) prompted us to investigate the basis of this phenomenon. Two-hundred seventy-eight *E. faecium* isolates of VanB phenotype from the period 1999 to 2010 obtained by NRCST were investigated by multilocus sequence typing (MLST) and multilocus VNTR analysis (MLVA). Localization, transferability, and partial structure of the *vanB*-carrying Tn*1549* transposon were studied by hybridization, PCR mapping, sequencing, and conjugation. VanB isolates almost exclusively represented hospital-associated *E. faecium*, with a significant shift from representatives of 17/18 lineage to 78 lineage after 2005. The *vanB* determinant, initially located mostly on transferable plasmids of the pRUM-, pLG1-, and pRE25-replicon types, later on was found almost exclusively on the host chromosome. Fifteen different plasmid and chromosomal insertion sites were identified, typically associated with single transposon coupling sequences, mostly not observed before. Our study demonstrates the significant change in the epidemiology of VanB-*E. faecium* in Poland, associated with the introduction and spread of the lineage 78 of the hospital-adapted *E. faecium*. These data point to the importance of the lineage 78 for the spread of vancomycin-resistance, determined by the *vanB* gene cluster, resulting in an increasing VRE prevalence in hospitals. This study also supports the scenario, in which representatives of the hospital-associated *E. faecium* independently acquire the *vanB* determinant de novo and spread within and among hospitals, concomitantly undergoing differentiation.

**Electronic supplementary material:**

The online version of this article (10.1007/s10096-018-3209-7) contains supplementary material, which is available to authorized users.

## Introduction

The importance of enterococci as etiologic agents of hospital-acquired infections (HAIs) is currently increasing [1], and common glycopeptide resistance among these bacteria is especially alarming [2]. Among two most ubiquitous *van* gene clusters, responsible for this phenotype, *vanA* confers resistance to both vancomycin and teicoplanin, and *vanB* typically determines resistance only to vancomycin [3]. The *vanB* cluster is predominantly associated with the Tn*1549-*type transposons [4], which may reside either on plasmids or on the bacterial chromosome [4–9]. During the initial steps of conjugative transfer of transposon, the staggered cleavage by the Int recombinase results in the formation of a circular intermediate, joined by a 5- to 6-bp sequence originating from the donor genome, termed a coupling sequence, which, after transposition, is found adjacent to the transposon termini in the recipient [7].

Among the two clinically most important enterococcal species, i.e., *Enterococcus faecalis* and *Enterococcus faecium*, the latter is particularly prone to the acquisition of antimicrobial resistance determinants, including *vanA* and *vanB* clusters (vancomycin-resistant *E. faecium*, VR*Efm*), resulting in increasing proportion of VR*Efm* among hospital *E. faecium* [10]. Concomitantly, an increase in the incidence of HAIs caused by *E. faecium* is observed [10, 11], likely due to the selection and worldwide dissemination of successful hospital-adapted clonal complex 17 (CC17) [12] that combines resistance to several antimicrobials with the enrichment in pathogenicity factors and increased epidemic potential. The Bayesian Analysis of Population Structure (BAPS) of the data obtained by multilocus sequence typing (MLST) demonstrated that CC17 may be divided into two subgroups corresponding to major lineages 17/18 and 78 [13].

The *vanB* gene was identified in *E. faecalis* at the beginning of the 1990s [14]*.* In Poland, the first VR*Efm* with *vanB2* was isolated in 1999 [15] followed by a growing VanB prevalence in 1999–2005 [16]. A further increase in VanB-VR*Efm* after 2005, noticed by the National Reference Centre for Antimicrobial Resistance and Surveillance (NRCARS), prompted us to investigate these important pathogens to better understand the factors underlying the spread of VanB-*E. faecium* in Poland.

## Materials and methods

### Bacterial isolates and antimicrobial susceptibility testing

Altogether, 278 non-repetitive isolates with the VanB phenotype received by the NRCARS during 1999–2010 from 36 centers in 22 cities were investigated. Fifty-eight VanB isolates from 1999 to 2005 were partly characterized previously [16]; of these, 56 were available and 222 isolates were received in 2006–2010. Twenty-seven and 48 isolates were obtained from invasive and non-invasive infections, respectively, and 201 from carriage; for two remaining isolates, the source was not reported. Antimicrobial susceptibility was tested using the broth microdilution method [17] and the Etest method for vancomycin, teicoplanin, and daptomycin (bioMerieux, Marcy l’Etoile, France). Results were interpreted following the European Committee on Antimicrobial Susceptibility Testing (EUCAST)-approved breakpoints [18] and the Epidemiological Cut-Offs (ECOFFs) (http://mic.eucast.org/Eucast2/, 6th November 2017, date last accessed).

### Detection of *vanB*, IS*16* and *esp*, and molecular typing

DNA was purified using the Genomic DNA Prep Plus kit (A&A Biotechnology, Gdynia, Poland) and *vanB*; IS*16* and *esp* were detected by PCR [19–21]. Multilocus VNTR (variable-number tandem repeat) analysis (MLVA) and MLST were performed as described [22, 23]; sequence types (STs) were assigned using the MLST database http://pubmlst.org/efaecium/ (6th November 2017, date last accessed). On the basis of eBURST analysis [24] of the whole MLST database (as of the 21st of April 2015), STs were included into CCs and lineages [13, 25].

### Analysis of Tn*1549*, insertion sites, and coupling sequences

The presence of *int*_Tn*1549*_ and ORF1_Tn*1549*_ was confirmed by PCR, and the *vanY-vanX* sequence in Tn*1549* was established using overlapping PCR and sequencing. The Tn*1549* insertion sites were identified by inverse-PCR (iPCR) [26] with *Bsp*143I (Fermentas, Lithuania). Primers targeting sequences adjacent to Tn*1549* were designed based on iPCR results. Sequences were analyzed with the Lasergene package (DNASTAR, MD, USA). Primer sequences are available upon request.

### Plasmid gene detection, S1 profiles, hybridization, and conjugation

Plasmid *rep* (*rep1*_pIP501_, *rep2*_pRE25_, *rep8*_pAM373_, *rep9*_pAD1_, *rep17*_pRUM_, *rep*_pMG1_, *rep*_pLG1_) and toxin-antitoxin systems (TAS) *axe-txe* and ω*-ε-ζ* were detected by PCR [26–29] with controls from our collection [28, 30]. For profiling of plasmids, DNA in agarose plugs was treated with S1 nuclease (Takara Bio, Japan), separated by pulsed-gel electrophoresis (PFGE) [31] and blotted onto Hybond-N+ (GE Healthcare, Buckinghamshire, UK). Hybridization was carried out using the Amersham ECL System (GE Healthcare). Transferability of vancomycin resistance was examined as described [32] with the recipient *E. faecium* strain 64/3.

### Statistics

The differences in distributions were evaluated by the chi-squared test, with a *p* value ≤ 0.05 considered significant.

### GenBank accession numbers

New sequences of the *vanY-vanX* region: A1-A6 (KC489780-KC489785), A9-A20 (KT003969-KT003980), B1 (KC489787), B2 (KT003981), D (KC489790), and E (KT003982); *rep17*_pRUM_ (KM014782), *rep*_pLG1_-1 (KM014783), and *rep*_pLG1_-2 (KM014784) were submitted to GenBank.

## Results

### Antimicrobial susceptibility phenotypes and clonal relationships of VanB-VR*Efm* in Poland

All isolates were analyzed by MLVA, yielding 23 different MTs; 13 non-typable isolates repeatedly yielded incomplete MLVA profiles (Table 1). The most prevalent MT159 (186 isolates, 83.0%) was observed solely since 2006. Eighty isolates from 2006 to 2010, representing all hospitals providing isolates and all MTs, were resistant to ciprofloxacin and ampicillin; 88.8 and 88.8% isolates showed high-level resistance to gentamicin (HLGR) and streptomycin (HLSR), respectively; 20.0% of isolates were resistant to tetracycline, which represented a significant decrease (*p* = 0.0002) after 2005 (61.5% [16]). All isolates were susceptible to linezolid, tigecycline, and daptomycin. STs of 26 VanB isolates from the period 1999–2005 were reported previously [16], and additionally 21 isolates from this group were analyzed by MLST, together with 80 representative isolates from the period 2006–2010, mentioned above, yielding altogether 23 STs, characteristic for 127 isolates. Except for ST74, all isolates belonged to lineages 17/18 and 78, and representatives of 78 lineage were frequently associated with MT159 (Table 1). All isolates carried *vanB* and IS*16*; *esp* was present in 98% isolates from 2006 to 2010, similarly to the earlier period [16]. Based on the combined MLVA and MLST results, no representatives of lineage 78 were observed before 2006; the first VanB isolate from lineage 78 occurred in 2006 and since 2007 isolates from this lineage became much more common, representing 89% of isolates from the period 2006–2010 (*p* < 0.001).

**Table 1:** Epidemiological and typing data, *vanY-vanX* region structure and Tn*1549* localization among VanB *E. faecium* in Poland, 1999–2010

A. Plasmid localization of Tn*1549*							
Variant name^a^	Centre (*n*)^a^	Year	ST (*n*)^b^	MT (*n*)^b^	Line age	*vanY-vanX* (*n*)^b^	*vanB*-plasmid representatives approximate size in kb; {plasmid-specific genes}; (*n*)^b^
CS_P1a	KRA2 (4)	2000 2000	381 (2) 384 (2)	264 (2) 326 (2)	17/18 17/18	A3	- 100 {*rep*2} - 160 {−}; 320 {*rep*2, *rep*_pLG1_, *axe-txe*} - 150 {*rep*_pLG1_} (2)
CS_P1b	WAW1 (4)	1999–2000 1999–2000	382 (2) 383 (2)	325 (2) 325 (2)	17/18 17/18	B1	- 100 {*rep*2, *axe-txe*} - 80 {*rep*2, *rep*_pLG1_, *axe-txe*} - 100 {*rep*2}; 240 {*rep*2, *rep*17, *rep*_pLG1_, *axe-txe*} - 60 {*rep*2, *rep*17, *rep*_pLG1_, *axe-txe*}; 90 {*rep*2, *rep*17, *rep*_pLG1_, *axe-txe*}
CS_P2	SZC2 (9)	2002 2005 2005 2005 2005	386 562 260 (2) 920 74	4 375 (4) 13 (2) 231 *nt*	17/18 17/18 17/18 17/18 S	C (10)	- 220 {*rep*2, *rep*17, *rep*_pLG1_, *axe-txe*} - 220 {*rep*17, *rep*_pLG1_, *axe-txe*} - 220 {*rep*17, *rep*_pLG1_} (2) - 80 {*rep*17, *rep*_pLG1_}; 220 {*rep*17, *rep*_pLG1_} - 240 {*rep*_pLG1_} - 300 {*rep*17, *rep*_pLG1_, *axe-txe*} - 220 {−} - 160 {*rep*_pLG1_}
CS_P3	POZ1 (1) WAW3 (1) WRO (3) ZGO (1) ZAB1 (1)	2003 2005 2005 2006 2007	202 18 440 17 279	1 1 7 4 3	17/18 17/18 17/18 17/18 17/18	A4 A4 A4 A9 A12	- 200 {*rep*2, *rep*17, *rep*_pLG1_, *axe-txe*} - 150 {−} - 150 {−} - 150 {−} *nd*
CS_P4	WAW1 (11)	2005 2005 2005 2005	17 387 (4) 384 384	4 (5) 50 (4) 325 326	17/18 17/18 17/18 17/18	D (11)	- 150 {−} - 150 {−} - 150 {−} - 150 {−}
CS_P5	WAW5 (1)	2005	387	376	17/18	A2	- 120 {*rep*2}
CS_P6a1	KSZ (2) WAW2 (1)	2010 2010	78 (2) 17	159 (2) 11	78 17/18	A20 A19	- 250 {*rep*2, *rep*17, *rep*_pLG1_} (2) ^c^HMW
CS_P6a2	KSZ (1)	2010	78	159	78	*nd*	- 250 {*rep*2, *rep*17, *rep*_pLG1_}
CS_P6b	POZ7 (6)	2008	78 (2)	159 (6)	78	A13, A14	- 250 {*rep*2, *rep*17, *rep*_pLG1_} (5)
CS_P7	LUB (3) POZ1 (2)	2009–2010 2009	78 (3) 192 (2)	159 (3) 159 (2)	78 78	A15	- 70 {*rep*2, *rep*17, *rep*_pLG1_, *axe-txe*} (2) - < 50 {*rep*2}, 270 {*rep*17, *rep*_pLG1_, *axe-txe*} - 80 {*rep*2, *rep*17, *rep*_pLG1_, *axe-txe*} (2)
B. Chromosomal localization of Tn*1549*							
Variant name^a^	Centre (*n*)^a^	Year	ST (*n*)^b^	MT (*n*)^b^	Lineage	*vanY-vanX* (*n*)^b^	
CS_C1	KRA1 (1) KON (13) KAL (1) POZ1 (1) POZ2 (2) KRA1 (1) POZ6 (1) POZ7 (1)	2003 2004 2004 2004 2005 2005 2006 2008	387 387 (13) 387 387 387 (2) 387 561 202	50 50 (3), nt (10) 50 376 50 (2) 50 50 1	17/18 17/18 17/18 17/18 17/18 17/18 17/18 17/18	A1 A1 A1 A1 A1 A1 A1 *nd*	
CS_C2	WAW1 (1)	2005	279 (1)	152	17/18	A8	
CS_C3	KKE (1) BYD (2) KSC (1) PLO (1) GWP (12) KSZ (56) POZ1 (20) POZ2 (48) POZ3 (5) POZ4 (7) POZ5 (2) POZ6 (5) POZ7 (4) ZGO (17) KIE (1) SZC2 WAW3 (1) WAW4 (1) ZAB2 (1) KAL (1) NWS (1) OBR (1) OLS (8)	2006 2007 2007 2007 2007–2008 2007–2010 2007–2009 2007 2010 2008–2009 2009 2010 2007–2008 2007–2010 2008 2008 2008 2008 2008 2010 2010 2010 2010	78 78, 856 78 78 78 (6), 192 17, 18, 267 78 (2) 64, 918 (2) 78 (3) 64, 267 (4), 857 78 (7) 78 78 78 78 (2) 78 (4) 382 78 (3) 78 78 (2) 78 78 78 78 78 78 78 (2), 192	12 299, 159 159 159 159 (12) nt, 1 (3), 7 159 (51) 1, nt (2) 159 (15); 334 (2) 1 (8), 296 159 (35), 250, 293 (3) 159 (5) 159 (7) 334 (2) 159 (5) 11, 159 (2), 291 50 159 (16) 159 159, 334 159 159 159 159 159 159 159 (8)	78 78 78 78 78 17/18 78 17/18 78 17/18 78 78 78 78 78 78 17/18 78 78 78 78 78 78 78 78 78 78	A2 A2 (2) A2 A2 A2 A1 *nd* A2 A2 A2 A2 A2 A2 A2 *nd* A11 *nd* A1 A1 A2 C E A2 A17 A2 A18 F (8)	
CS_C4	PRZ (1) PSZ (1)	2004 2005	279 561	231 231	17/18 17/18	A5 A6	
CS_C5	LUB (1)	2008	78	159	78	F	
CS_C6	ZAB1 (1)	2007	18	7	17/18	A10	
CS_C7	OLS (2)	2009	78	159 (2)	78	B2, C	
CS_C8	SZC1 (1)	2010	192	159	78	A16	

^a^*BYD*, Bydgoszcz; *GWP*, Gorzów Wlkp.; *KAL*, Kalisz; *KIE*, Kielce; *KKE*, Kędzierzyn-Koźle; *KON*, Konin; *KRA*, Kraków; *KSC*, Kościerzyna; *KSZ*, Koszalin; *LUB*, Lublin; *NWS*, Nowa Sól; *OBR*, Oborniki; *OLS*, Olsztyn; *PLO*, Płock; *POZ*, Poznań; *PRZ*, Przasnysz; *PSZ*, Pszczyna; *SZC*, Szczecin; *WAW*, Warszawa; *WRO*, Wrocław; *ZAB*, Zabrze; *ZGO*, Zielona Góra; numbers adjacent to these abbreviations indicate hospitals in a city; number of isolates from a hospital given in brackets

^b^Number of isolates given in brackets if different from one

^c^*HMW*, high-molecular weight DNA band, a presumable integration of plasmid into chromosome; *S*, singleton; *nt*, non-typable; *nd*, not determined

### Diversity of the *vanY-vanX* region in Tn*1549*

**Fig. 1:**
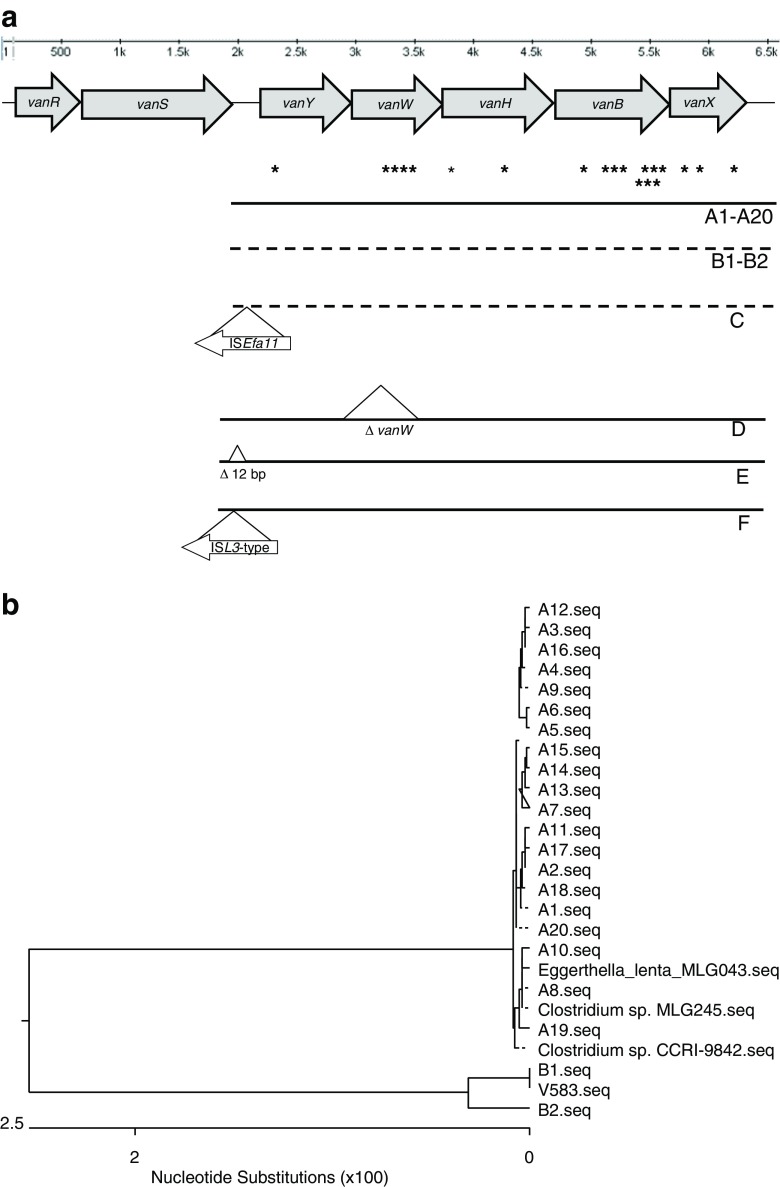
Diversity of *vanY-vanX* region among VanB-VR*Efm* in Poland, 1998–2010. **a** Structure of the region, distribution of single-nucleotide polymorphisms (marked by asterisks) among A-type variants, and localization of deletions and ISs. **b** Similarity tree of nucleotide sequences of A- and B-type variants and sequences from the V583 strain of *E. faecalis* and isolates of *E. lenta* and *Clostridium* spp

All isolates were positive for ORF1_Tn*1549*_ and *int*_Tn*1549*_. Sequencing of the *vanY-vanX* region (encompassing genes *vanY*, *vanW*, *vanH*, *vanB*, *vanX*; Fig. 1a) revealed 26 variants among 57 isolates, representing all centers and STs within a center. The most numerous group included A1-A20 variants, differing only by single-nucleotide polymorphisms (SNPs) at 21 nucleotide positions and highly similar to the corresponding region in *Clostridium* spp. and *Eggerthella lenta* (Fig. 1b). The A-type variants were characteristic for 48 of investigated isolates from 31 centers, and associated with 14 STs and 16 MTs. The B variants differed from the A-type by several SNPs and 6-bp insertion between *vanS-vanY*. They were 99% identical to the variant reported for the V583 [33]. An insertion of the IS*Efa11* between *vanS-vanY* in B-type yielded C variants (Fig. 1a). The D-, E-, and F-types represented probable derivatives of an A-type, with a deletion encompassing the nt 12-799 of *vanW*, an 11-bp deletion upstream *vanY*, and insertion of IS*L3* between *vanS* and *vanY*, respectively. All *vanB* genes represented the *vanB2* variant [34].

### Analysis of Tn*1549* insertion sites and coupling sequences

To determine Tn*1549* insertion sites, selected isolates were analyzed by iPCR and thus obtained sequences were used to search GenBank and to design primers specific for a genetic neighborhood of Tn*1549*. These primers were used to screen the whole collection, revealing 15 insertion sites and 14 coupling sequences in total (Table 2). For two isolates, the coupling sequence could not be established due to the fact that sequences resulting from iPCR had no homologs in GenBank. Typically coupling sequences were identical in a given insertion site, with an exception of CS_P1a/CS_P1b in *aacA-aphD* and CS_P6a/CS_P6b in *citH*. The most prevalent coupling sequence, CS_C3 (198 isolates from 24 centers in 16 cities) was associated with 16 MTs and 14 STs. The first CS_C3 isolate was observed in 2006 (Table 1B).

**Table 2:** Insertion sites and coupling sequences of Tn*1549*-type transposons in VanB *E. faecium* in Poland, 1999–2010

Variant name^a^	Number of isolates	Flanking target sequence (20 bp)^b^	CS	Tn*1549*		CS	Flanking target sequence (20 bp)^b^	Insertion region
				Left end	Right end			
								Plasmid (GenBank hits)
CS_P1a	4	TTAGTACTAAATTTTGTTTT_676_	–	AAAATTTTAG	ATATAATTTT	GTTTT	_675_AAAATGTATTCATTATTAAC	*aacA-aphD* (LT598665)
CS_P1b	4	TTAGTACTAAATTTTGTTTT_676_	–	AAAATTTTAG	ATATAATTTT	TATAT	_675_AAAATGTATTCATTATTAAC	*aacA-aphD* (LT598665)
CS_P2	10	ATTTATCTTGCTGATTATTT_79_	TTGAGG	AAAATTTTAG	ATATAATTTT	–	_80_TTTCTCAAAACCATACTAAA	*cadD* (CP011829)
CS_P3	7	GAGAAAGTCGAATTATTTTT_89_	ATTTGG	AAAATTTTAG	ATATAATTTT	–	_90_AACACAAAAATTAGCAGAGG	Ef_aus00233 plasmid 3 ORF (nt 493,131–49,077; LT598665)
CS_P4	11	TTATTAATTATTTTTGATCT	–	AAAATTTTAG	ATATAATTTT	GGTAG	AAAAATTAGCTTAACAAATA	Intergenic in p63-1 (CP019989) (AL021_14715-AL021_14720)
CS_P5	1	AATAGCATATTTTTCTGTGC	*nd*	AAAATTTTAG	ATATAATTTT	*nd*	CAATCTCAAAATTTCGTTGA	Unknown (no GenBank hits)
CS_P6a1	2	GGGCTAAAATGCTTGGTTTT_912_	GTACAT	AAAATTTTAG	ATATAATTTT	–	_913_TATCCCTAAAAATATCGAAA	*citH* Aus0085 plasmid 1 (CP006621)
CS_P6a2	1	GGGCTAAAATGCTTGGTTTT_912_	*GTACAT*	AAAATTTTAG	ATATAATTTT	*GTACAT*	_919_TAAAAATATCGAAAAAGGTG	*citH* Aus0085 plasmid 1 (CP006621)
CS_P6b	6	GGGCTAAAATGCTTGGTTTT_912_	TTATGA	AAAATTTTAG	ATATAATTTT	–	_913_TATCCCTAAAAATATCGAAA	*citH* Aus0085 plasmid 1 (CP006621)
CS_P7	5	CTGTTGCAAAGTTTTAAATA	–	AAAATTTTAG	ATATAATTTT	TTATGA	AAAGAAAAAATCCCTTACGG	intergenic in pTT39_p3 (CP023426) (*repB*_pseudogene-IS*6*)
								Chromosome^c^
CS_C1	21	TTCTAGCAGCTTTTATCGAA	–	AAAATTTTAG	ATATAATTTT	CCAATT	AAAACTTAGCATCAGCGACG	Intergenic (AFK60264-AFK60265)
CS_C2	1	ACTTCATTGCTTTTTAAATC_406_	–	AAAATTTTAG	ATATAATTTT	CACTA	_405_ACAACTGATATCCTTATACT	AFK59023
CS_C3	198	CTAGAAAAGGCCCAGCTTTT_843_	TGGCTA	AAAATTTTAG	ATATAATTTT	–	_842_TGCATAAAAGTTTGTGCGAG	AFK58314
CS_C4	2	CCACAAATAGAGTAAATTTT	ATCGT	AAAATTTTAG	ATATAATTTT	–	AGAATAAAATTTTAAAAAGG	Intergenic (AFK10635-AFK10636)
CS_C5	1	TGTATAATGAGAAAAATATT_677_	ATAGAA	AAAATTTTAG	ATATAATTTT	–	_678_AAAGGAAAATTTTGTCGATT	AFK58216
CS_C6	1	ATAGAGTAAATTTACAAATT	*nd*	AAAATTTTAG	–	*nd* ^d^	*nd* ^d^	Unknown (no GenBank hits)
CS_C8	2	GTGGATTTGATGTTATAAAA	–	AAAATTTTAG	ATATAATTTT	TTATAT	AAAAATTTCTCATTTTTGGC	Intergenic (IS*6770*_AFK57968)
CS_C9	1	CTTCTAAAAAATTTTCATTT_225_	–	AAAATTTTAG	ATATAATTTT	CATTT	_227_AAAAAACAACATCTGCGCAA	AFK58870

Duplicated CS italicized

*nd*, not determined

^a^*CS*, coupling sequence; *P*, plasmid integration site; *C*, chromosomal integration site

^b^For CS_P5 and CS_C6 sequences adjacent to the transposon termini are provided

^c^Hits corresponding to the DO genome of *E. faecium*

^d^No amplification product in the inverse-PCR

### Analysis of Tn*1549* localization, plasmidome composition, and *vanB* transferability

Seventy-eight isolates were analyzed by S1/PFGE-hybridization with the *vanB* probe (Table 1 and Supplementary Fig. 1). These isolates represented all observed variants of coupling sequence and hospital centers; additional isolates from the same center were included in the case of isolates showing plasmid localization of *vanB*. In the case of 39 isolates with coupling sequence C1-C8, *vanB* hybridized with a band of high-molecular weight, consistent with transposon insertion within chromosomal sequences and 39 isolates showed hybridization with plasmids from ~ 30 to ~ 310 kb in size; in five isolates, *vanB* was located on two plasmids. These hybridization studies and iPCR/PCR-based analyses of coupling sequences were consistent with the chromosomal localization of Tn*1549* for 227 isolates (81.6%) and plasmid localization for 50 isolates (18.0%); in a single case, a presumable integration of plasmid into chromosome was observed (variant CS_6a1 from WAW2). Isolates with the plasmid localization of *vanB* were much more prevalent among early VR*Efm*, i.e., from 1999 to 2005 (61% of these isolates) compared to the isolates collected from 2006 to 2010 (0.7% of these isolates, *p* < 0.001). Among isolates with the plasmid localization of *vanB*, *rep17*_pRUM_ was found among 49 isolates, followed by *rep*_pLG1_, *rep2*_pRE25_, *rep1*_pIP501_, *rep*_pMG1_, and *rep9*_pAD1_ (42, 35, 32, 27, and 5 isolates, respectively). Thirty-one and 12 of these isolates carried *axe-txe* and ω*-ε-ζ*), respectively. S1-PFGE/hybridization analyses revealed that 29, 23, and 22 plasmids hybridized with the *rep*_pLG1_, *rep17*_pRUM_, and *rep2*_pRE25_ probes, respectively (Table 1A). In several cases, a single plasmid was associated with two or three *rep* genes. Sixteen *vanB*-plasmids hybridized with the *axe-txe* probe; among them, 13 co-hybridized with *rep*_pLG1_ and 12 with *rep17*_pRUM_, respectively. Nine plasmids did not hybridize with any of the four probes used. Sequencing revealed a low variability of *rep* genes within this group that included two, one, three, one, and three variants of *rep1*_pIP501_, *rep2*_pRE25_, *rep17*_pRUM_, *rep*_pMG1_, and *rep*_pLG1_, respectively. Among 50 isolates with the plasmid localization of *vanB*, 43 isolates (86.0%) were able to transfer vancomycin resistance while conjugation experiments involving 32 representative isolates with various chromosomal localizations of *vanB* were negative in 29 cases.

## Discussion

The first VanB-VR*Efm* was detected in Poland in 1999 [15], and our study investigated the VanB epidemiology during the following 12 years. Considering a relatively moderate incidence of VR*Efm* in Poland during this period (e.g., in 2010 amounting to 7.8% of invasive infections [http://ecdc.europa.eu/en/publications/Publications/1111_SUR_AMR_data.pdf.pdf; 6th November 2017, date last accessed]), it may be assumed that our collection reasonably well reflected the epidemiological situation in Polish hospitals. Although initially VanA represented the major VR*Efm* phenotype in Poland [16, 35], after 2006, the NRCARS recorded an increasing number of VanB-VR*Efm*, affecting several hospitals. The current global epidemiology of VR*Efm* shows considerable differences, with VanA predominant in Europe and the USA [36], and VanB constituting over 80% of invasive VR*Efm* in Australia [37]. A recent rise of VanB-*E. faecium* has been reported in Germany [8]. Nearly all isolates in our study belonged to the hospital *E. faecium*, since 2006 with the predominant role (89%) of lineage 78. VanB-VR*Efm* belonging to this lineage were responsible for recent outbreaks in Germany, Sweden, and Australia [38], and representatives of lineage 78 played a role in *vanA* dissemination in Polish hospitals [34].

While the structure of Tn*1546*, harboring *vanA* shows a high variability [34, 39], the *vanY-vanX* region in the *vanB* gene cluster appeared to be less divergent. In the studied collection, the A-type showed the highest prevalence, with variants very similar or identical to these found in *E. faecium* in Australia, France, and Taiwan [40–42], and in the pMG2200 plasmid of *E. faecalis* [5]. Importantly, the A variants are also present in gut anaerobes such as *Clostridium* spp. and *E. lenta* [26, 40], a presumable reservoir of Tn*1549-*type transposons. Genomic analyses of VanB-*E. faecium* and concomitantly isolated *vanB-*positive gut anaerobes indicated the epidemiological significance of de novo acquisition of Tn*1549* by hospital-adapted *E. faecium* [7, 41]. The B-type characteristic for the first vancomycin-resistant *E. faecalis* V583 strain [33], to our knowledge, has not been reported in *E. faecium* so far. The presence of ISs targeting the *vanS-vanY* intergenic region (resulting in C- and F-types), was observed also elsewhere [43]. Such variability of *vanB* clusters may be useful in analyses of suspected VRE outbreaks. For example, plasmid-located D-type was found in isolates representing various MTs and STs from the WAW1 hospital (Table 1A). Thus, a spread of a stable ~ 150 kb conjugative plasmid of undetermined replicon type, harboring this specific variant of the *vanB* cluster was most likely responsible for the outbreak. Similarly, although isolates from SZC2 differed both in the clonal composition and *vanB-*associated plasmidome, C-type was detected in all these isolates (Table 1A), indicating extensive plasmid recombination during an outbreak. Until now, more detailed knowledge of plasmids carrying *vanB* in *E. faecium* remains limited [38]. In our study, *vanB*-plasmids represented mostly the *rep*_pLG1_, *rep17*_pRUM_, and *rep2*_pRE25_ replicons, similarly to the situation observed for *vanA-*plasmids in Poland [34]. The original pLG1 contained the complete *vanA* gene cluster [44] and plasmids with this *rep* were responsible for an increase of HLGR among *E. faecium* in Norway [45] but, to our knowledge, *vanB-*plasmid of the *rep*_pLG1_ type has not yet been reported. The second observed *rep* type, *rep17*_pRUM_ was involved in a multicenter VanB outbreak in Sweden [46] and in the HLGR spread in Norway [45]. The *rep*_pLG1_ and *rep17*_pRUM_ genes frequently occurred together and in combination with the *axe-txe*, characteristic for plasmids with these replicons [34, 45, 46]. Plasmids harboring *vanB* were typically transferable by conjugation and during outbreaks (e.g., in KRA2, WAW1 and SZC2) were associated with diverse clonal backgrounds. Such plasmid dissemination was additionally accompanied by presumable recombination/co-integration events, resulting in the observed variability of *vanB-*plasmids. A similar dynamics was observed also for *rep17*_pRUM_-type *vanA-*plasmids [30]. Recombination/co-integration likely contributed to the association of *vanB* with more than a single *rep*, observed in the current study and characteristic for *E. faecium* plasmids in general [30, 34, 38, 45]. Two plasmid-located genes, *aacA-aphD* and *citH*, showed the integration of Tn*1549* with different coupling sequences and might represent transposon integration hotspots. Such hotspots were indeed observed for *E. faecium* [7].

Isolates with plasmid-borne *vanB* prevailed until 2006, and later this determinant showed usually a chromosomal localization. This change occurred in parallel with the emergence and spread of lineage 78. The predominantly chromosomal localization of the *vanB* cluster in lineage 78 was observed recently also in Germany and Australia [7, 8, 40]. Two variants of coupling sequences, CS_C1 and CS_C3, were associated with two most numerous groups of isolates (Table 1). Twenty-one isolates with the CS_C1 variant, present in 17/18 lineage and A1 type of the *vanY-vanX* region, showed multicenter distribution over 2003–2008. These isolates showed some divergence of their STs/MTs, which may be explained by a transfer of transposon-containing region to a new clonal background [8] and/or exchange of other genomic regions, leading to formation of new STs/MTs [9]. An even more complex epidemiological situation was associated with isolates harboring the CS_C3 variant. This particular group appears to be the main contributor to the increasing proportion of VanB among VR*Efm* and general increase of prevalence of VRE in Poland and was responsible for extensive outbreaks, e.g., in KSZ and POZ2 hospitals. With the exception of a single isolate with CS_C5, which shared a coupling sequence and insertion site with several ST192 isolates from Germany [8], none of the remaining coupling sequences showed identity to coupling sequences described elsewhere [7–9]. This finding is consistent with proposed independent de novo acquisition of Tn*1549* [7].

This study provides an analysis of VanB-*E. faecium*, performed on a country level and over an extensive period of time. We demonstrate a significant change both in the clonal background as well as localization of Tn*1549-*type transposons, carrying *vanB* genes. Our study supports the role of lineage 78 of the hospital-adapted *E. faecium*, presumably acquiring de novo the *vanB* determinant, followed by spread and differentiation of certain strains as a major factor beyond the current increasing prevalence of VanB-VR*Efm* in Polish hospitals.

## Electronic supplementary material

Supplementary Fig. 1 Exemplary results of Southern hybridization of PFGE of S1-digested total DNA of VREfm isolates with plasmid localization of *vanB* using the following probes: *vanB*, rep_pLG1_, rep17_pRUM_, and rep2_pRE25_, as indicated on the right. Plasmids co-hybridizing with the *vanB* probe and *rep* probes are indicated by arrows. Lambda Ladder PFG Marker (New England BioLabs, UK) was used as a molecular weight standard, with approximate fragment sizes provided on the left. (PPTX 1435 kb)
