# Supplementary material for: The changing epidemiology of VanB Enterococcus faecium in Poland
Source: Eur J Clin Microbiol Infect Dis. 2018 Feb 13;37(5):927–36. doi: 10.1007/s10096-018-3209-7 (PMC5916999; doi:10.1007/s10096-018-3209-7)
Supplement: Supplementary file 1 — Exemplary results of Southern hybridization of PFGE of S1-digested total DNA of VREfm isolates with plasmid localization of vanB using the following probes: vanB, reppLG1, rep17pRUM, and rep2pRE25, as indicated on the right. Plasmids co-hybridizing with the vanB probe and rep probes are indicated by arrows. Lambda Ladder PFG Marker (New England BioLabs, UK) was used as a molecular weight standard, with approximate fragment sizes provided on the left. (PPTX 1435 kb) [file 10096_2018_3209_MOESM1_ESM.pptx]

## Slide 1
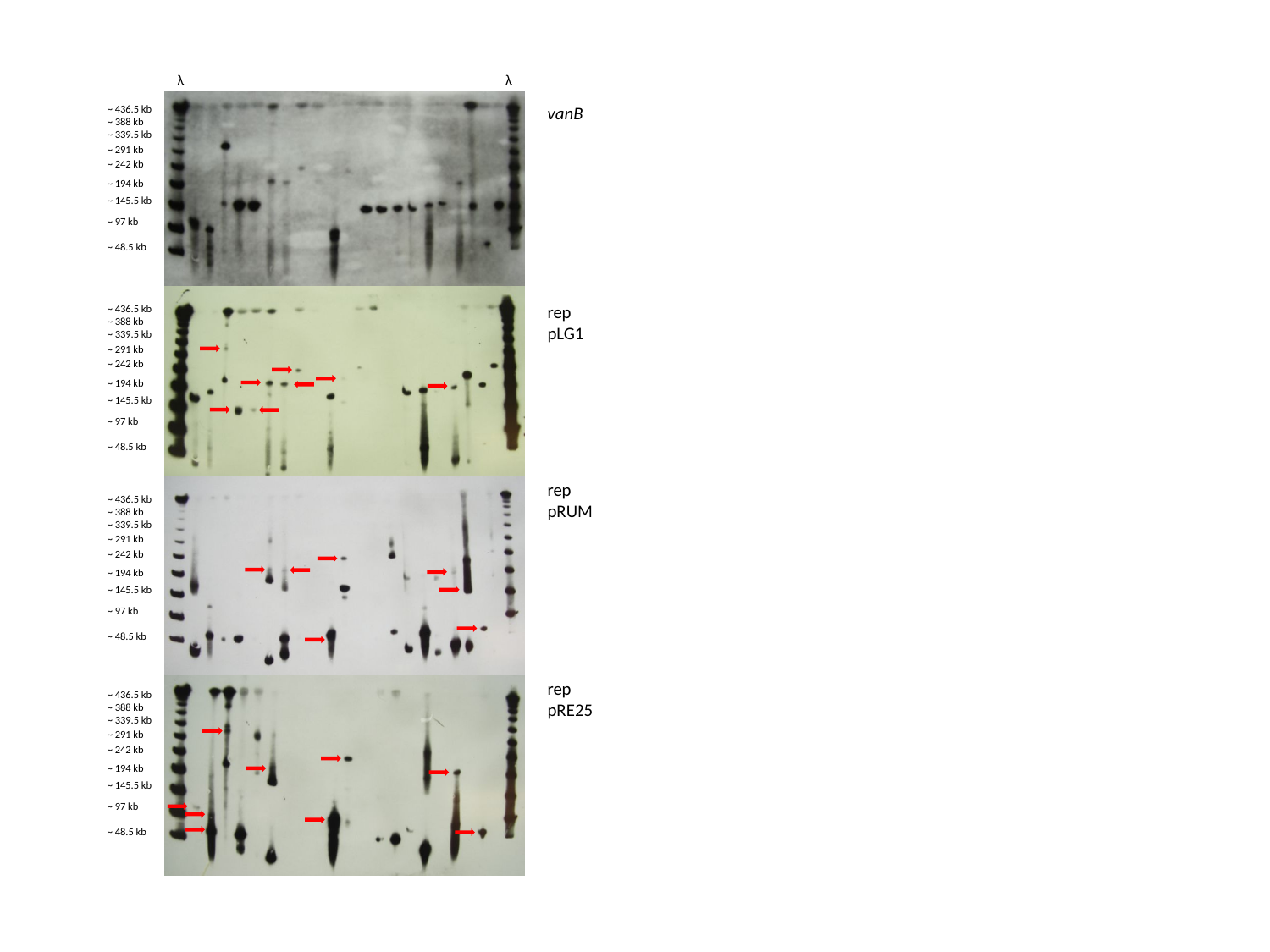

λ
λ
~ 436.5 kb
~ 388 kb
~ 339.5 kb
~ 291 kb
~ 242 kb
~ 194 kb
~ 145.5 kb
~ 97 kb
~ 48.5 kb
vanB
rep pLG1
rep pRUM
rep pRE25
~ 436.5 kb
~ 388 kb
~ 339.5 kb
~ 291 kb
~ 242 kb
~ 194 kb
~ 145.5 kb
~ 97 kb
~ 48.5 kb
~ 436.5 kb
~ 388 kb
~ 339.5 kb
~ 291 kb
~ 242 kb
~ 194 kb
~ 145.5 kb
~ 97 kb
~ 48.5 kb
~ 436.5 kb
~ 388 kb
~ 339.5 kb
~ 291 kb
~ 242 kb
~ 194 kb
~ 145.5 kb
~ 97 kb
~ 48.5 kb
